# Neuromuscular Plasticity in a Mouse Neurotoxic Model of Spinal Motoneuronal Loss

**DOI:** 10.3390/ijms20061500

**Published:** 2019-03-26

**Authors:** Rosario Gulino, Nunzio Vicario, Maria A. S. Giunta, Graziana Spoto, Giovanna Calabrese, Michele Vecchio, Massimo Gulisano, Giampiero Leanza, Rosalba Parenti

**Affiliations:** 1 Laboratory of Neurophysiology, Department of Biomedical and Biotechnological Science, Section of Physiology, University of Catania, Catania 95123, Italy; 2 Laboratory of Cellular and Molecular Physiology, Department of Biomedical and Biotechnological Sciences, Section of Physiology, University of Catania, Catania 95123, Italy; nunziovicario@unict.it (N.V.); alessandragiu94@gmail.com (M.A.S.G.); grazianaspoto@gmail.com (G.S.); giovanna.calabrese@unict.it (G.C.); 3 Rehabilitation Unit, “AOU Policlinico Vittorio Emanuele” and Department of Biomedical and Biotechnological Sciences, Section of Pharmacology, University of Catania, Catania 95123, Italy; michele.vecchio@unict.it; 4 Laboratory of Synthetic and Systems Biology, Department of Drug Sciences, University of Catania, Catania 95125, Italy; m.gulisano@unict.it; 5 Laboratory of Neurogenesis and Repair, Department of Drug Sciences, University of Catania, Catania 95125, Italy; gpleanza@unict.it

**Keywords:** neurodegeneration, spinal cord, gastrocnemius muscle, CTB-Saporin, neuronal plasticity, AMPA receptor, motoneuron, astrocyte

## Abstract

Despite the relevant research efforts, the causes of amyotrophic lateral sclerosis (ALS) are still unknown and no effective cure is available. Many authors suggest that ALS is a multi-system disease caused by a network failure instead of a cell-autonomous pathology restricted to motoneurons. Although motoneuronal loss is the critical hallmark of ALS given their specific vulnerability, other cell populations, including muscle and glial cells, are involved in disease onset and progression, but unraveling their specific role and crosstalk requires further investigation. In particular, little is known about the plastic changes of the degenerating motor system. These spontaneous compensatory processes are unable to halt the disease progression, but their elucidation and possible use as a therapeutic target represents an important aim of ALS research. Genetic animal models of disease represent useful tools to validate proven hypotheses or to test potential therapies, and the conception of novel hypotheses about ALS causes or the study of pathogenic mechanisms may be advantaged by the use of relatively simple *in vivo* models recapitulating specific aspects of the disease, thus avoiding the inclusion of too many confounding factors in an experimental setting. Here, we used a neurotoxic model of spinal motoneuron depletion induced by injection of cholera toxin-B saporin in the gastrocnemius muscle to investigate the possible occurrence of compensatory changes in both the muscle and spinal cord. The results showed that, following the lesion, the skeletal muscle became atrophic and displayed electromyographic activity similar to that observed in ALS patients. Moreover, the changes in muscle fiber morphology were different from that observed in ALS models, thus suggesting that some muscular effects of disease may be primary effects instead of being simply caused by denervation. Notably, we found plastic changes in the surviving motoneurons that can produce a functional restoration probably similar to the compensatory changes occurring in disease. These changes could be at least partially driven by glutamatergic signaling, and astrocytes contacting the surviving motoneurons may support this process.

## 1. Introduction

Severe loss of spinal and bulbar motoneurons (MNs) is a characteristic feature of different MN diseases, including amyotrophic lateral sclerosis (ALS) and spinal muscular atrophy (SMA). ALS is a rare disease with an incidence of about 2 per 100,000 in the Caucasian population [1]. The majority of ALS patients have a spinal onset of the disease, characterized by focal muscle weakness starting either distally or proximally in the upper or lower limbs. Bulbar onset may occur in some patients showing dysarthria or dysphagia, followed by limb weakness. Paralysis of skeletal muscles is progressive and leads to death due to respiratory failure within 2–5 years after diagnosis [2].

Despite considerable efforts in both basic and translational research in ALS, the causes of the disease are still unknown and no effective treatment is available. Nevertheless, a huge amount of data has improved the knowledge on ALS pathogenesis [3,4,5], suggesting a variety of complex pathological mechanisms that challenge the vision of ALS as a simple MN disease [6]. Indeed, many authors indicate that ALS is a multi-system disease caused by a network failure instead of a degenerative process restricted to a specific neuronal population [6,7]. Although MN loss is the critical hallmark of ALS given the specific vulnerability of these cells to a hostile tissue microenvironment, other cell populations are involved in disease onset and progression, including glial and muscle cells [5,8], but understanding the specific role of each cell population and their crosstalk requires further investigation. Glutamate excitotoxicity [4], oxidative stress [4,8], and the role of different genetic alterations, including mutations of the genes encoding for Cu/Zn superoxide dismutase 1 (SOD1), TAR-DNA binding protein (TDP-43), fused in sarcoma (FUS), and C9orf72, have been documented in animal models as well as in patients affected by both familial and sporadic ALS [4], but the interplay between these hallmarks, as well as their relationship with environmental factors, are still to be elucidated. Moreover, given the multifactorial cause of this disease, there is an evident need for other animal models, since the available genetic models are unable to fully elucidate the underlying pathophysiology and potential exploitable mechanisms. We suggest that the use of reductionist models, although not strictly mimicking the disease, could help in understanding the single parts of this complex pathological process.

It seems probable that pathogenesis could have a focal origin in a region of the central nervous system where genetic and environmental factors may contribute to create a toxic milieu, where microglia and astrocytes probably share a role in excitotoxic insults to neurons and synapses [1,8]. In this context, toxic effects of mutated SOD1, TDP-43, or other altered cell-derived products by known or still unknown mutations could participate in loss of function, local degeneration, and also in the spread of pathological insults and disease progression [4]. To unravel this intricate mechanism, all these elements should be investigated independently in order to reveal players that may represent the trigger of the pathogenic process. In this complex scenario, less attention has been devoted to the plastic properties of the degenerating motor system. Indeed, several studies demonstrated that abnormalities in MNs and spinal cord (SC) circuitries are present far earlier than disease onset in genetic mouse models of ALS [9,10]. This means that the whole neuromuscular system is capable of compensatory plastic changes that maintain the system’s efficiency despite the MN loss, but these attempts to balance the progressive degeneration invariably fail when degeneration reaches a given severity threshold. Therefore, as symptoms only appear when MN degeneration is rather severe, it seems reasonable that the plastic properties of the affected tissue should be studied in more detail and can possibly be used as therapeutic targets, together with a much earlier diagnosis, with the aim of slowing-down or hopefully stopping the progressive degeneration.

We have previously generated a model of selective MN degeneration by injecting cholera toxin-B saporin (CTB-Sap) [11,12] into the gastrocnemius muscle (GM) of wild-type mice [13]. This represents a reductionist model of focal depletion of SC MNs [12,13,14], where the effects of selective MN removal could be studied in experimental conditions that are markedly different than those in genetic models of disease. Considering that synaptic failure is one of the most recent and trusted hypotheses of ALS pathogenesis [6], this model could represent a promising tool for studying plastic changes after MN degeneration in the absence of other confounding factors that are present in other more complex animal models, as well as in patients. Here, we have produced a unilateral SC lumbar MN depletion by injecting CTB-Sap into the mouse GM. Then, motor impairment and subsequent recovery were studied and spinal and muscle pathology was characterized. Finally, cholinergic activity, glial reaction, and fast excitatory synaptic transmission were evaluated by quantifying choline acetyltransferase (ChAT), glial fibrillary acidic protein (GFAP), and AMPA (α-amino-3-hydroxy-5-methyl-4-isoxazole propionic acid)-type glutamate receptors subunits’, GluR1 and GluR4, expression levels, respectively, in relation to plasticity and functional recovery.

## 2. Results

### 2.1. Behavioral Impairment Induced by CTB-Sap Lesion

**Figure 1:**
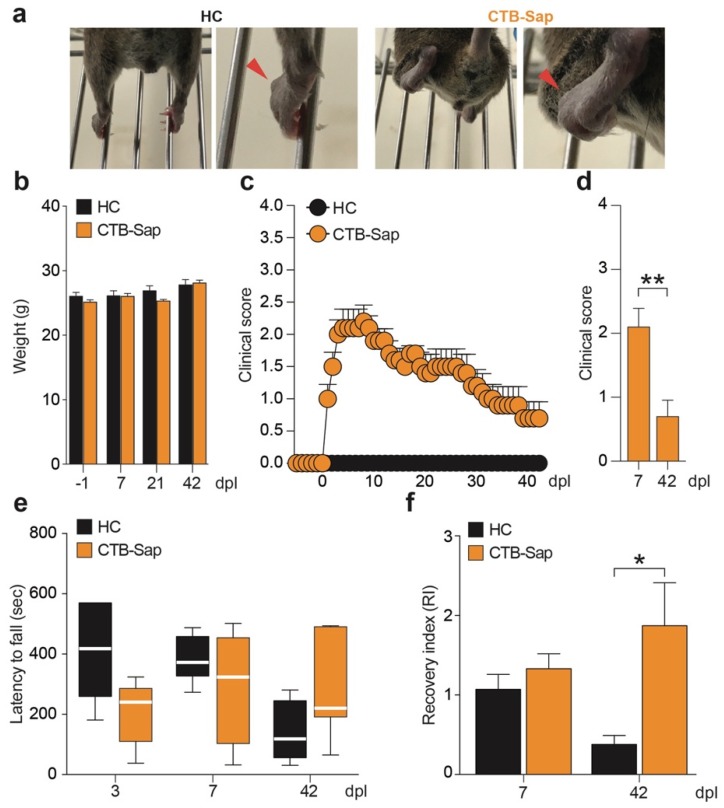
Behavioral impairment induced by selective spinal MN ablation. (**a**) Representative pictures of hindlimbs of HC and CTB-Sap lesioned mice; red arrows indicate the capability in HC or the lack of toes spreading of the left hindlimb of CTB-Sap mice; (**b**) HC and CTB-Sap mice body weight at −1, 7, 21 and 42 day post lesion (dpl); data are shown as mean ± SEM; (**c**,**d**) clinical score during the time course of motor impairment (**c**) and bar plots at 7 and 42 dpl (**d**); data are shown as mean ± SEM; ** *p*-value < 0.01; unpaired *t*-test. (**e**) Latency to fall expressed in sec on rotarod test at 3, 7, and 42 dpl in HC and CTB-Sap lesioned mice; data are expressed as standard box-and-whiskers plot in which the central-line represents the median and the upper and lower bounds are min and max value; (**f**) recovery index (RI) of HC and CTB-Sap mice at 7 and 42 dpl over index at 3 dpl. Data are expressed as mean ± SEM; * *p*-value < 0.05; one-way ANOVA and Holm-Sidak’s multiple comparisons test.

In order to evaluate the effects of a unilateral loss of MNs, the behavioral deficits of CTB-Sap-lesioned vs. healthy control (HC) mice were evaluated. Despite a major impairment of the CTB-Sap injected left hindlimb (Figure 1a), animals were able to move and explore and they did not show any sign of distress or significant weight loss, as compared to HC mice (Figure 1b).

Then, the functional impairment of the left hindlimb was evaluated in a time-course manner by scoring subjects for hindlimb posture and motor function, thus detecting the early signs of impairment as early as 1 day post lesion (dpl) with a significant worsening at 7 dpl (Figure 1c). Interestingly, a spontaneous amelioration was seen at 42 dpl as compared to the clinical score at 7 dpl (0.7 ± 0.25 at 42 dpl vs. 2.1 ± 0.29 at 7 dpl, *p*-value = 0.0068; Figure 1d). Moreover, despite no significant differences being observed in the rotarod performance vs. HC mice (Figure 1e), CTB-Sap mice showed a significantly higher recovery index (RI) at 42 dpl (1.87 ± 0.54 CTB-Sap vs. 0.38 ± 0.11 HC, *p*-value = 0.0318; Figure 1f).

**Figure 2:**
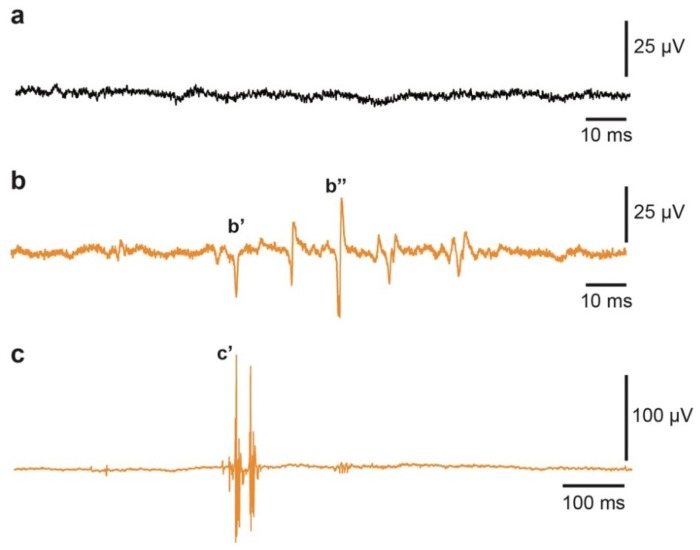
Abnormal spontaneous activity in CTB-Sap-induced acute GM denervation. (**a**) EMG recording of spontaneous activity in HC left GM at 42 dpl; (**b**) positive sharp waves (**b’**) and fibrillation (**b’’**) in CTB-Sap left GM at 42 dpl; (**c**) multiplet motor unit neuromyotonic discharges (**c’**) and irregular bursts of discharges in denervated GM.

Electromyography (EMG) recordings of the spontaneous GM activity in HC GM muscle (Figure 2a) versus CTB-Sap lesioned GM revealed that lesioned mice exhibit typical signs of denervation, including positive sharp waves (psw; Figure 2b–b’) and fibrillations (Figure 2b–b’’). Since these are not the exclusive EMG signs of neuromuscular disorders, we analyzed the EMG profile in more depth, finding clear signs of increased excitability of peripheral motor axons (i.e., neuromyotonia), characterized by doublet, triplet, or short bursts of high frequency discharge (Figure 2c–c’), thus indicating that a number of motor units among those spared by the lesion likely developed hyperexcitability with typical spontaneous discharges.

### 2.2. Anatomical Changes of Denervated Muscle

**Figure 3:**
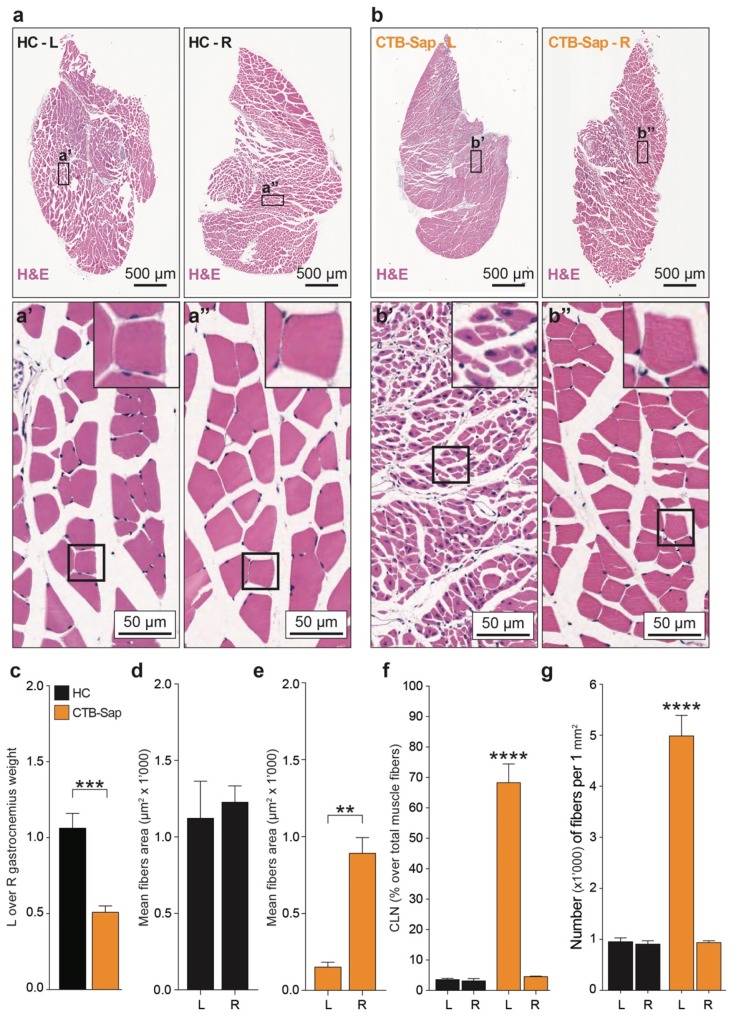
CTB-Sap-induced muscular changes. (**a**,**b**) Representative pictures of left (L) and right (R) GMs of HC (**a**) and CTB-Sap mice (**b**) and high magnification images of L (**a’**) and R (**a’’**) GM of HC and L (**b’**) and R (**b’’**) GM of CTB-Sap mice. (**c**) L over R GM weight in HC and CTB-Sap lesioned mice; data are shown as mean ± SEM; *** *p*-value < 0.001; unpaired *t*-test; (**d**,**e**) mean fiber area of L and R GM of HC (**d**) and CTB-Sap (**e**) mice; data are shown as mean ± SEM; ** *p*-value < 0.01; unpaired *t*-test; (**f**) centrally located nuclei (CLN) quantification in L and R GMs of HC and CTB-Sap mice; data are shown as percentage of CLN over total muscle fibers ± SEM; **** *p*-value < 0.0001; one-way ANOVA and Holm-Sidak’s multiple comparisons test; (**g**) number of fibers (×1000) per 1 mm^2^ area in L and R GMs of HC and CTB-Sap mice; data are mean ± SEM; **** *p*-value < 0.0001; one-way ANOVA and Holm-Sidak’s multiple comparisons test.

As CTB-Sap mice were still symptomatic at 42 dpl, a morphological analysis of isolated left and right GMs of both HC (Figure 3a–a’’) and CTB-Sap (Figure 3b–b’’) mice was carried out. A significant reduction of the ratio between the left and right muscle weight was observed in CTB-Sap compared to HC mice (*p*-value = 0.0008; Figure 3c). In particular, the average left muscle weight in CTB-Sap mice was 0.083 ± 0.01 g and the average right muscle weight was 0.169 ± 0.02 g. No significant difference was found in left vs. right GM weight in HC mice (0.214 ± 0.03 g left vs. 0.200 ± 0.01 g right; Figure 3c).

From a morphological point of view, a striking difference could be observed between the left GM (CTB-Sap-injected; Figure 3b’) and either the right GM (not injected; Figure 3b’’) of lesioned mice or the muscles of HC mice (Figure 3a). In particular, lesioned muscles showed a strong reduction of the fiber diameter that, together with the 50% decrease of the muscle weight, may suggest evident atrophy. This effect of the lesion was quantified by measuring the cross-sectional area of muscle fibers, thus showing no differences in left vs. right mean values in HC (*p*-value > 0.05; Figure 3d), and, conversely, a substantial reduction of the average cross-sectional area in injured (left) muscles compared to the intact (right) counterparts (152.7 ± 7.3 μm^2^ vs. 891.3 ± 25.54 μm^2^, *p*-value < 0.01; Figure 3e).

Finally, the percentage of myofibers exhibiting centrally located nuclei (CLN) was quantified as an index of myopathy, but also to determine which myofibers were undergoing repair in response to acute denervation. As expected, a small proportion of CLN was found in intact muscles (i.e., left and right GM of HC mice and right GM of CTB-Sap mice; Figure 3f), whereas the analyses of lesioned GMs revealed a robust increase of CLN (Figure 3f). This evidence, together with the strong increase of the number of fibers (about 5-fold; Figure 3g), suggests a regenerative process within the denervated muscle.

**Figure 4:**
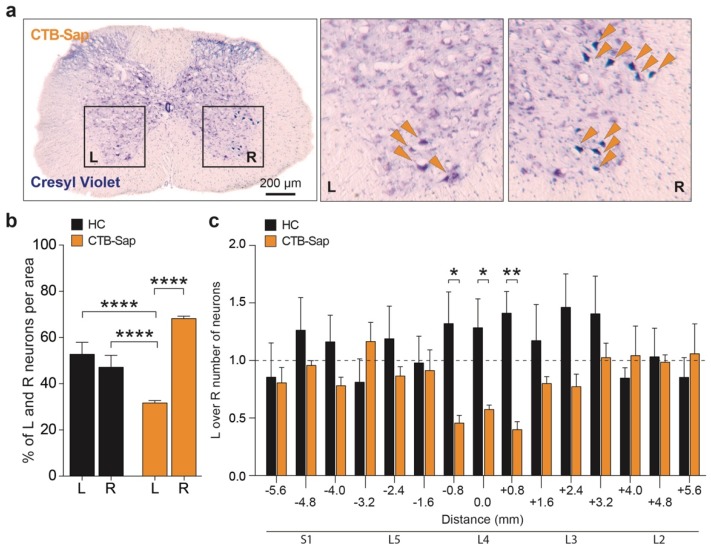
Permanent ablation of spinal MNs in Rexed lamina IX of CTB-Sap injected mice. (**a**) Representative pictures of cresyl violet staining on a 20 μm axial section of the lumbar portion of the SC of CTB-Sap mice; orange arrows in the inserts indicate MN bodies, thus showing their reduced number in L compared to R side; (**b**) quantifications of the percentages of total neurons in the L and R side of the lumbar portion of the SC in HC and CTB-Sap mice; data are shown as mean ± SEM; **** *p*-value < 0.0001; one-way ANOVA and Holm-Sidak’s multiple comparisons test; (**c**) quantification of MNs in the SC (S1–L2) of HC and CTB-Sap mice; data are shown as mean ± SEM; * *p*-value < 0.05; ** *p*-value < 0.01; one-way ANOVA and Holm-Sidak’s multiple comparisons test.

To confirm the ablation of the MN pool in the SC, a quantification of the cresyl violet positive cells was performed in the left and right Rexed lamina IX of HC and CTB-Sap mice SCs. CTB-Sap lesion induced a selective depletion of MNs in the left SC ventral horn (31.71 ± 1.77% of total MNs) as compared to the contra-lateral right side (68.29 ± 1.77% of total MNs, *p*-value < 0.0001 vs. CTB-Sap left side, *p*-value < 0.0001) and to HC (52.82 ± 2.92% left and 47.18 ± 2.92% right, *p*-value < 0.0001 vs. CTB-Sap left side, *p*-value < 0.0001; Figure 4a–b). Quantification of spinal MNs in the lumbar tract of the SC reveals that the unilateral injection of CTB-Sap selectively removed MNs in the L4–L5 segments, as expected given the distribution of the MN pool innervating the GM (Figure 4c).

### 2.3. Plasticity Mechanisms in the SCs of CTB-Sap-Lesioned Mice

**Figure 5:**
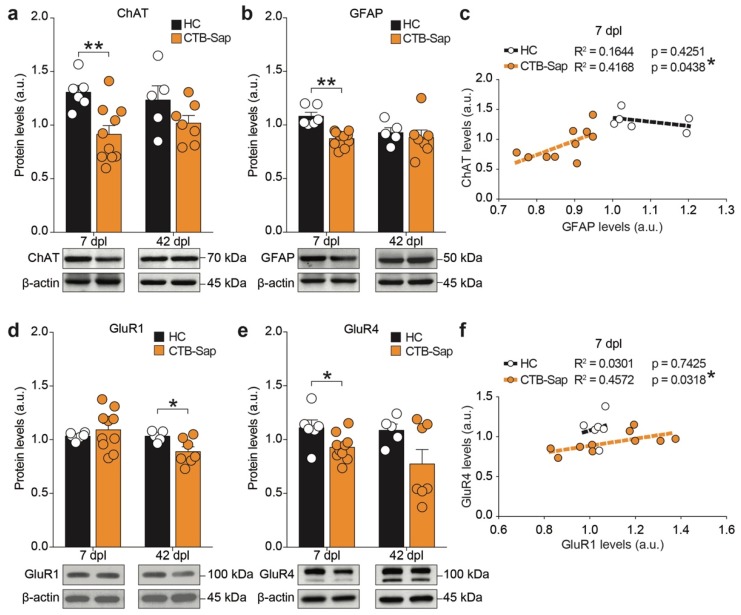
Spinal plasticity in CTB-Sap lesioned mice involves AMPA receptor subunits, GluR1 and GluR4. (**a**,**b**) Quantification and representative blots of ChAT (**a**) and GFAP (**b**) protein levels at 7 and 42 dpl in HC and CTB-Sap lesioned mice; data are shown as mean ± SEM; each dot represents a single subject (*n* = 6 HC, and *n* = 10 CTB-Sap mice); ** *p*-value < 0.01, unpaired *t*-test; (**c**) linear regression analysis of ChAT and GFAP levels in HC and CTB-Sap mice; * *p*-value < 0.05; (**d**,**e**) quantification and representative blots of GluR1 (**d**) and GluR4 (**e**) protein levels at 7 and 42 dpl in HC and CTB-Sap lesioned mice; data are shown as mean ± SEM; each dot represents a single subject (*n* = 6 HC, and *n* = 10 CTB-Sap mice); * *p*-value < 0.05, unpaired *t*-test; (**f**) linear regression analysis of GluR1 and GluR4 levels in HC and CTB-Sap mice; * *p*-value < 0.05.

Given the permanent depletion of a large number of MNs innervating the GM in CTB-Sap lesioned mice, followed by compensatory mechanisms, the lumbar tract of the SC was analyzed to quantify the levels of ChAT, as an index for cholinergic activity in the SC, and GFAP, as an astroglial marker. Western blot quantification revealed a significant reduction of ChAT in lesioned mice as compared to HC in the lumbar SC at 7 dpl (Figure 5a). Importantly, ChAT expression levels in the lesioned SC were similar to HC levels at 42 dpl, thus suggesting a spontaneous compensatory increase (Figure 5a). Similarly, the analysis of GFAP expression in the total protein content of the lumbar SC revealed a reduction of GFAP levels at 7 dpl, followed by a spontaneous recovery at 42 dpl (Figure 5b).

In order to analyze the relationship between spinal MNs and astrocytes, a linear regression analysis of ChAT and GFAP levels was carried out. The results showed that in HC mice there is no significant correlation between these markers at both 7 dpl (*p*-value = 0.4251, *R*^2^ = 0.1644, Figure 5c) and 42 dpl (*p*-value = 0.3771, *R*^2^ = 0.2628). Vice versa, CTB-Sap mice exhibit a linear correlation between GFAP and ChAT at 7 dpl (*p*-value = 0.0438, *R*^2^ = 0.4168, Figure 5c), which was not significant at 42 dpl (*p*-value = 0.6151, *R*^2^ = 0.0543), indicating a potential relationship between the surviving ChAT-positive neurons and astroglial cells in the SC carrying a partial MN depletion.

In an effort to find the potential players involved in plasticity, as well as in the interplay between neurons and glial cells, the expression levels of the AMPA receptor subunits, GluR1 and GluR4, were measured. We observed near-normal levels of GluR1 in the early phase of MN depletion (*p*-value = 0.4626 vs. HC; Figure 5d) and a significant reduction at 42 dpl (*p*-value = 0.0317 vs. HC; Figure 5d). Interestingly, a significant reduction of GluR4 was seen at 7 dpl (*p*-value = 0.0277 vs. HC; Figure 5e) followed by a restoration at 42 dpl (*p*-value = 0.0919 vs. HC; Figure 5e).

### 2.4. GluR1 and GluR4 Are Involved in Neuronal Plasticity upon Selective Spinal MN Depletion

**Figure 6:**
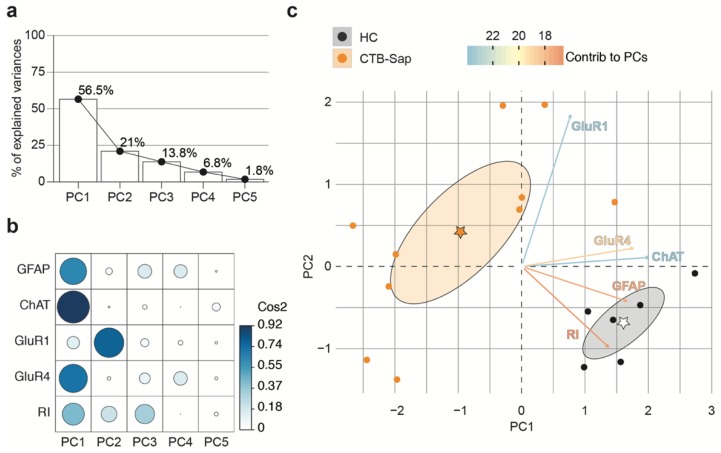
The association between behavioral and molecular outcomes at 7 dpl assessed by principal components analysis (PCA). (**a**,**b**) Scree plot of PCs (PC1–PC5) and the percentage of GluR1, GluR4, ChAT, and GFAP, and the recovery index (RI) explained variance (**a**) and quality of representation of the variables on the factor map expressed as square cosine (Cos2); (**c**) PCA biplot of GluR1, GluR4, ChAT, GFAP, and RI data for *n* = 10 CTB-Sap lesioned mice and *n* = 6 HC mice; key colored arrows represent the variables’ contribution to the PC; stars indicate the mean points of groups, and confidence ellipses are also shown.

In order to analyze the multivariate effect of proteins levels and the rotarod test upon acute depletion of MNs, a principal component analysis (PCA) was performed on CTB-Sap lesioned vs. HC mice, by integrating the individual levels of ChAT, GFAP, and AMPA subunits at 7 dpl with that of the rotarod latency to fall on the same subjects. We first integrated our data generating principal components and found that the principal components (PCs) PC1 and PC2 explain a total of 77.5% of the variance at 7 dpl (Figure 6a).

**Figure 7:**
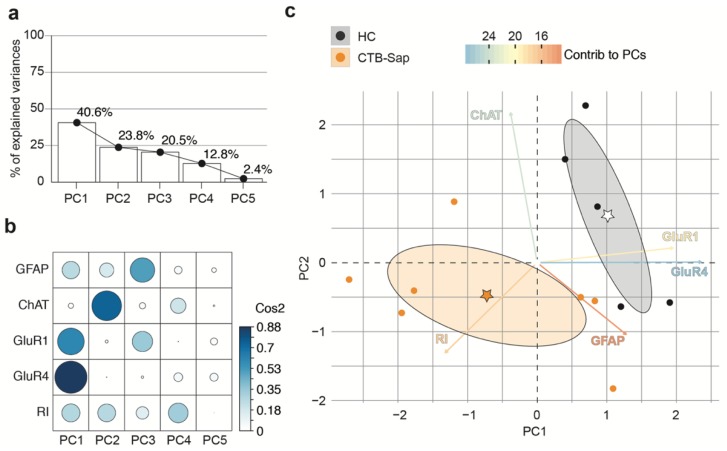
The association between behavioral and molecular outcomes at 42 dpl assessed by principal components analysis (PCA). (**a**,**b**) Scree plot of PCs (PC1–PC5) and the percentage of GluR1, GluR4, ChAT, and GFAP, and the recovery index (RI) explained variance (**a**) and quality of representation of the variables on the factor map expressed as square cosine (Cos2); (**c**) PCA biplot of GluR1, GluR4, ChAT, GFAP, and RI data for *n* = 7 CTB-Sap lesioned mice and *n* = 5 HC mice; key colored arrows represent the variables’ contribution to the PC; stars indicate the mean points of groups, and confidence ellipses are also shown.

To analyze the quality of the representation of variables in PC1 and PC2, we performed a square cosine–square coordinates (cos2) analysis of PCA (Figure 6b). The results showed that GFAP, ChAT, and GluR4 were highly represented in PC1 (GFAP cos2 = 0.6312, ChAT cos2 = 0.9199, GluR4 cos2 = 0.7090) and that GluR1 and RI influenced both PC1 (GluR1 cos2 = 0.1369, RI cos2 = 0.4302) and PC2 (GluR1 cos2 = 0.7771, RI cos2 = 0.2196; Figure 6b). As such, we next assessed the differences between the HC and CTB-Sap groups using PC1 and PC2 and we found that GluR1 and ChAT were the most influencing variables in the CTB-Sap vs. HC comparisons (Figure 6c). Then, the same analysis was performed using the dataset relative to mice sacrificed at 42 dpl, thus finding that PC1 and PC2 explain a total of 64.4% of the variance (Figure 7a). The quality analysis of the representation of variables in PCA showed that GluR1 and GluR4 highly contribute to PC1 (GluR1 cos2 = 0.6609, GluR4 cos2 = 0.8763). GFAP and RI variables were represented in both PC1 (GFAP cos2 = 0.2570, RI cos2 = 0.2747) and PC2 (GFAP cos2 = 0.1704, RI cos2 = 0.2679) and, finally, ChAT influenced mainly PC2 (ChAT cos2 = 0.7437) with low representation in PC1 (ChAT cos2 = 0.0231).

**Figure 8:**
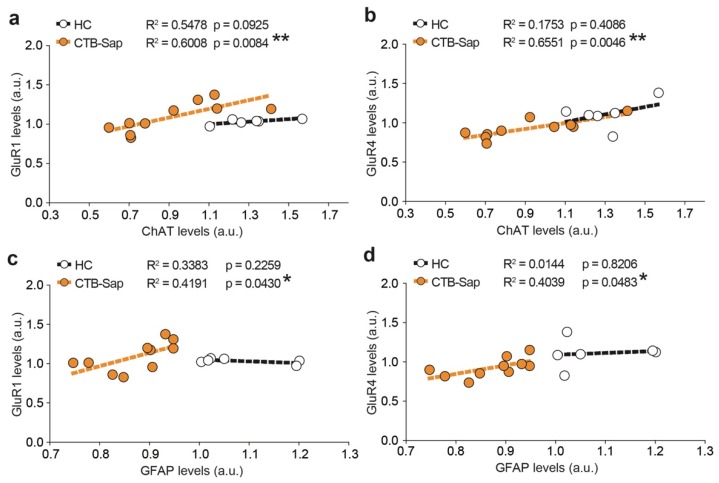
Significant correlations between spinal MNs and astrocytes and AMPA subunits, GluR1 and GluR4. (**a**,**b**) Linear regression analysis of GluR1 (**a**) or GluR4 (**b**) levels and ChAT levels; ** *p*-value < 0.01. (**c**,**d**) Linear regression analysis of GluR1 (**c**) or GluR4 (**d**) levels and GFAP levels; * *p*-value < 0.05.

In order to confirm the above results obtained by analyzing the whole dataset, linear regression and correlation was performed between the GluR1/GluR4 expression levels and either those of ChAT (i.e., neurons, Figure 8a–b) or GFAP (i.e., astrocytes, Figure 8c–d).

The results revealed strong linear correlations between the expression levels of GluR1 and ChAT (*R*^2^ = 0.6008, *p*-value = 0.0084; Figure 8a), GluR4 and ChAT levels (*R*^2^ = 0.6551, *p*-value = 0.0046; Figure 8b), GluR1 and GFAP (*R*^2^ = 0.4191, *p*-value = 0.0430; Figure 8c), and GluR4 and GFAP (*R*^2^ = 0.4039, *p*-value = 0.00483, Figure 8d), indicating that, upon neuronal depletion, GluR1 and GluR4 AMPA receptor subunits are involved in the spinal changes of surviving MNs and astrocytes.

## 3. Discussion

In recent years, a number of studies have focused on both ALS patients and animal models of human neurodegenerative diseases, showing that abnormalities in SC circuitries and MNs, including the disconnection of neuromuscular junctions [6,15], and even the death of a relevant number of MNs, appear far earlier than the onset of ALS symptoms [9,16,17,18,19,20]. This implies that a number of plastic changes are likely to take place within the neuromuscular system as an attempt to compensate for the progressive loss of motor units. In our view, unraveling these complex processes should be one of the priorities of the fundamental research on neurodegenerative diseases, together with finding the mechanisms underlying the spread of MN degeneration. The efficient targeting of these processes coupled with efficient methods of early diagnosis would increase the probability of slowing down the disease progression and hopefully promoting repair.

Herein, we found that after CTB-Sap injection into the left GM, the posture and locomotion of the ipsilateral hindlimb were dramatically impaired during the acute phase of the disease, as animals were unable to walk normally and could not grip, but a significant recovery was seen by both the rotarod test and clinical score at 6 weeks after toxin injection, despite a strong MN depletion in the left side of the lumbar SC and signs of denervation of the left GM were still present. Indeed, EMG recordings have demonstrated that this model of MN degeneration closely mimics many of the signs of denervation and increased excitability of spared motor units usually present in denervated muscles, including fibrillations, positive sharp waves, and neuromyotonic discharges [21]. Taken together, these findings suggest that the functional damage induced by selective MN depletion in the SC is stable over time, inducing neurological and behavioral impairment that was likely compensated, at least partially, by spontaneous mechanisms of plasticity occurring among the spared motor units. Denervated GMs appeared atrophic, as demonstrated by the reduction of muscle weight and the average cross-sectional area of muscle fibers, but, interestingly, the number of muscle fibers per area was increased and a high number of CLNs were counted in muscle sections, thus suggesting an attempt of muscle regeneration [22,23,24,25]; this may have been, at least partially, allowed by the selectivity of CTB-Sap to target MNs [11,12]. These findings are also interesting given that an increased cross-sectional area of muscle fibers has conversely been observed in ALS patients and animal models [26,27], as well as in SMA animal models, even in the presence of many atrophic muscle fibers [28].

In our view, this discrepancy between ALS and our CTB-Sap model confirms that ALS is not a cell-autonomous MN disease and that different cell types, including muscle fibers, are independently involved in the pathological processes [29,30,31], whereas the muscle modifications observed in the CTB-Sap model are produced by denervation, as no primary defects are expected to be present in muscles. So, the comparison between different animal models will provide important information about the mechanisms under evaluation.

However, muscular plasticity is probably not sufficient to support recovery and any motor amelioration should have been driven also by functional modifications within the spared motor units. After CTB-Sap injection, the loss of lumbar MNs was also paralleled by a significant downregulation of ChAT expression levels at one week after the lesion. It has been previously shown that about 80% of the acetylcholine released within the SC is not from supraspinal sources [32], but originates from the local MN activity, whereas the remaining could be linked to other cholinergic synaptic activity within the SC, as also demonstrated by a number of studies exploring the spinal cholinergic system [33,34,35]. Thus, the observed decrease of ChAT after CTB-Sap lesion may be caused in part by the loss of MNs, and in part by the consequent disruption of the surrounding spinal circuitry. These events could together result in a reduction of neural activity that in turn could be responsible for the motor impairment. Indeed, despite the dramatic MN loss, ChAT expression recovered up to control levels at 6 weeks after the lesion. Moreover, at this time-point, the hindlimb function appeared also improved, despite the MN depletion. Therefore, an increased activity in the spared MNs and the surrounding spinal circuits, probably as a result of an increased synaptic efficacy, is likely to be responsible for both the recovery of ChAT expression and the restoration of motor performance. Such an increase in synaptic efficacy has been already demonstrated after SC injury or disease [36,37,38] and also in the CTB-Sap model, where modifications of synapsin I expression were observed several weeks after the lesion [37,39,40,41]. Supraspinal control of movement may also be involved in such complex circuitry rearrangement [42,43,44,45,46]. Indeed, similar modifications in spinal or supraspinal circuitry were documented in ALS animal models and patients [16,46,47,48,49,50], but their role is unclear and the involvement in the whole pathogenic mechanism is complex, so their study in simpler models would be helpful.

Interestingly, the reduction of ChAT expression was accompanied by a similar down-regulation of GFAP in lesioned animals, with statistically significant correlations between their expression levels present in lesioned, but not in control SCs. Similar to ChAT expression, the reduction of GFAP levels was restored at 42 days post-lesion. Taken together, these results suggest that the CTB-Sap lesion has probably triggered a degeneration of astrocytes contacting the degenerating MNs. Conversely, the lack of difference in GFAP expression between lesioned and control animals at six weeks after the lesion suggests a compensatory reaction within the astrocyte population that sustains the functioning and compensatory changes of the spared MNs. This is an intriguing hypothesis given the emerging theories about the role of astrocytes in producing a hostile microenvironment surrounding MNs, which is probably responsible for a non-cell-autonomous degeneration of MNs in ALS [51,52,53,54,55]. Other evidence suggesting that our model could probably mimic such a mechanism is the modification of the expression levels of the AMPA glutamate receptors subunits, GluR1 and GluR4, in CTB-Sap lesioned animals, which was differentially modulated during the time-course of the disease. In particular, PCA and linear regressions helped us to highlight links between MNs and AMPA subunits, thus suggesting that this reduction may be due, at least in part, to the loss of the glutamatergic synapses contacting the degenerating MNs. Unexpectedly, the expression levels of both GluR1 and GluR4 were also found to correlate with those of GFAP in lesioned, but not in healthy controls. This suggests that compensatory changes to the homeostatic regulation of glutamatergic synapses by astrocytes surrounding the surviving MNs could be part of the process underlying the plasticity of the motor system, which in turn is responsible for the observed functional recovery after the initial disruption of SC circuitry induced by MN loss. Therefore, the glutamatergic system has undoubtedly a central role in MN diseases, not only for its involvement in excitotoxic insults, which are part of the pathogenic mechanisms of ALS [56,57,58,59], but also as a part of plasticity mechanisms occurring during the pre-symptomatic disease stage, when the motor system is still functioning despite the already dramatic depletion of the MN population.

We propose that although the known *in vivo* models of disease including, among others, those carrying the mutated human *sod1*, *tdp-43*, and *C9orf72* genes, represent useful tools to validate the proven hypotheses or to test candidate drugs, the creation of novel hypotheses about the etiology or the dissection of the pathogenic mechanism of MN diseases, may benefit from relatively simpler *in vivo* models, being able to mimic only a small and well-defined series of disease features. In close similarity to our model, other authors have proposed rodent models, where defined, small subpopulations of MNs have been removed by using CTB-Sap injection to mimic different aspects of ALS pathogenesis separately from each other. For instance, intrapleural injection can be used to mimic respiratory dysfunctions [60,61,62], and also to study respiratory plasticity after motoneuron depletion, without the concurrent participation of other confounding factors that are present in ALS models and patients [62]. Similarly, injecting CTB-Sap in the tongue muscles represents a simple model of dysphagia, which is another typical symptom of bulbar ALS [61]. The comparison between such a reductionist model and a more complex disease model would help to define the links between causes and their effects, avoiding the inclusion of too many confounding factors in the experimental setting. Here, by using a neurotoxic model of selective spinal MN depletion, we have proven that the skeletal muscle undergoes atrophy and displays spontaneous electromyographic activity, also observed in human neurodegenerative disorders, though observed differences in muscle fiber morphology suggest that some muscular effects of disease may be primary effects instead of being caused by denervation. Moreover, we found that plastic changes in the spared MNs can produce a functional restoration probably similar to the compensatory changes occurring during the pre-symptomatic stages of MN diseases. Then, we showed that at least some of these changes could be driven by glutamatergic signaling, and that astrocytes contacting the surviving MNs may support this process. Obviously, these changes are unable to halt the progression of ALS, and this is probably due to the pathological milieu affecting the surviving motoneurons, which invariably die during disease progression. Use of the CTB-Sap model would help in unraveling at least some of the mechanisms involved in this pathological environment. For instance, an ongoing study of this model of MN depletion would explain how the MNs’ death per se, together with changes in MN excitability, would affect the function of spared MNs in a long-term scenario.

## 4. Materials and Methods

### 4.1. Subjects and Experimental Design

All experiments were performed in accordance with the principle of the Basel Declaration as well as to the European Communities Council directive and Italian regulations (EEC Council 2010/63/EU and Italian D.Lgs. no. 26/2014). Moreover, the study was conducted in accordance with the recommendations of the local committee for animal welfare (OPBA, University of Catania, Via Santa Sofia 97, Catania, Italy); the protocol was approved by OPBA and by the Italian Ministry of Health (auth. no. 1133/2016-PR). All efforts were made to replace, reduce, and refine the use of laboratory animals. Experiments were performed on 8–12 weeks old male 129S1/SvImJ (Jackson Laboratory) weighing 25.6 ± 0.4 g. Animals were kept at constant temperature (23–25 °C) under a 12/12 h light/dark cycle with ad libitum access to food and water. A total number of 38 animals were used in this study. Animals were randomly assigned to the HC group (*n* = 16) or the CTB-Sap lesioned group (*n* = 22) that were allowed to survive for either 7 (HC, *n* = 6; CTB-Sap, *n* = 10) or 42 dpl (HC, *n* = 10; CTB-Sap, *n* = 12).

### 4.2. Neurotoxic Ablation of Spinal MNs

In order to induce MN depletion, mice were anesthetized with isoflurane (4% induction, 1.5% maintenance), and received 2 injections of the retrogradely transported, ribosome-inactivating toxin, CTB-Sap (Advanced Targeting Systems, San Diego, CA, USA), into the medial and lateral left GM, as previously described [24,39,63]. Each injection contained 3 μg CTB-Sap in 2 μL PBS (phosphate buffered saline).

### 4.3. Behavioral Tests

A daily evaluation of the hindlimb posture and locomotion was performed by two separate observers blind to the treatment using the following clinical score, adapted from a protocol used for phenotypic neurological score of ALS animal models [64,65]: 0 = healthy; 1 = collapse or partial collapse of leg extension towards the lateral midline during the tail suspension test; 2 = toes curl under at least twice during walking of 30 cm or any part of the foot is dragging along the cage bottom/table; 3 = rigid paralysis or minimal joint movement, foot not being used for generating forward motion; 4 = mouse cannot straighten itself within 30 s after being placed on either side.

Body weights were recorded at −1, 7, 21, and 42 dpl and the rotarod test was administered at 3 dpl and then at the end of the survival period (7 or 42 dpl). The rotarod test was carried out as previously described [13], in order to evaluate balance, muscle strength, and coordination. Briefly, mice were pre-trained on an automated 5-lanes rotarod device (Ugo Basile, Gemonio, Varese, Italy), fitted with a rotating cylinder (3.5 cm diameter) to which rubber bands were applied to improve grip, following a fixed speed training protocol. Then, after 2 min of running at a constant low speed (20 r.p.m.), animals were tested for a maximum of 10 min with an increasing speed, ranging from 20 to 60 r.p.m., and the performance was evaluated by measuring the latency to fall (in seconds). Each animal performed two trials per session, with 15 min resting between trials, and the values were averaged. For each animal, the ratio between late and early rotarod performance (latency to fall, in seconds) was calculated and is indicated as RI.

### 4.4. Electromyography

For EMG, animals were anesthetized with isoflurane as above and subjected to EMG recordings at 42 dpl. GMs were examined by a portable two-channel EMG device (Myoquick, Micromed S.p.A., Mogliano Veneto, Treviso, Italy) using 1 bipolar concentric needle electrode inserted in the GM and 1 grounded electrode. The spontaneous electrical activity of the muscle was recorded and then analyzed offline using System PLUS Evolution software by Micromed S.p.A. (Mogliano Veneto, Treviso, Italy).

### 4.5. Ex Vivo Tissue Pathology (Muscles and SCs)

At 42 dpl, HC (*n* = 5) and CTB-Sap (*n* = 5) animals were anesthetized with an intraperitoneal injection of ketamine 10 mg/mL and xylazine 1.17 mg/mL, and transcardially perfused with 0.5 M ethylene diamine tetra acetic acid (EDTA, Sigma-Aldrich s.r.l., Milano, Italy) in normal saline, followed by ice cold 4% paraformaldehyde in PBS (pH = 7.4). SCs and muscle were isolated and post-fixed in the same fixative solution at 4 °C for 24 h.

For SC histology, samples were washed in PBS and cryoprotected in 30% sucrose in PBS at 4 °C for 72 h. Samples were embedded in optimum cutting temperature (OCT) medium, snap frozen in liquid nitrogen, and stored at −80 °C until use. Frozen blocks were sectioned using a cryostat (Reichert-Jung 2800, Leica Microsystems, Buccinasco, Milano, Italy) with a microtome blade (Patho Cutter, Erma Inc., Tokyo, Japan), and 20 μm-thick axial sections were mounted on slides and stored at −80 °C until use.

For quantification of MNs in the Rexed lamina IX, SC sections were stained with cresyl violet, coverslipped and acquired using a ScanScope CS/GL (LRi Imaging AB, Lund, Sweden) microscope, and analyzed using ImageJ software (NIH, Bethesda, MD, USA). The number of MNs was obtained from the left and right side of the SC and reported as the percentage over the total number of MNs in the L4–L5 tract or as the left over right ratio. In all counts, only cell profiles with unambiguous motoneuronal morphology and size were considered.

For muscle pathology, the GMs were isolated, weighted, and fixed overnight in 4% paraformaldehyde. Tissue samples were processed using an automated tissue processor (Leica ASP300S, Leica Biosystems, Buccinasco, Milano, Italy). Briefly, muscles were dehydrated with an increasing concentration of ethanol, then immersed in xylene and incubated with paraffin (Bio-Optica, Milano, Italy) at 60 °C for 1.5 h. GMs were then embedded in paraffin blocks and 3 µm-thick axial sections were cut using an automated microtome (Leica RM225, Leica Biosystems).

For the hematoxylin and eosin (H&E) staining, tissue was processed as previously described with some modifications [66,67]. Briefly, sections were deparaffinized in xylene and rehydrated in decreasing concentrations of ethanol solutions. Nuclei were counterstained with Mayer’s hematoxylin (Bio-Optica) for 5 min and then differentiated in running tap water for 10 min. Cytoplasm was stained with alcoholic eosin solution (Eosin Yplus alcoholic solution—Bio-Optica) for 3 min. Sections were then dehydrated with increasing concentrations of ethanol solutions and then in xylene. Slides were coverslipped with synthetic mounting medium (EUKITT, Hatfield, PA, USA) and images were acquired using a ScanScope CS/GL (LRi Imaging AB) microscope and analyzed using ImageJ software. For quantification, 3 regions of interest (ROI) of a consistent size per muscle/mice were randomly selected and the area of all muscle cells was measured using ImageJ software. For each muscle fiber, the position of the nucleus was evaluated and the total number of nuclei in the ROI was counted and reported as a percentage of CLN over the total number of muscle fibers. CLN were considered as nuclei located in the inner cytoplasm versus normal muscle cells with the nucleus anchored at the periphery. All quantifications were performed by investigators blind to the treatment groups.

### 4.6. Immunoblotting

The lumbar portion of the SC of HC and CTB-Sap mice was isolated and homogenized in 1× RIPA lysis buffer (10 μL/mg tissue; abcam, Cambridge, UK), supplemented with a cocktail of protease inhibitors (Sigma-Aldrich s.r.l., Milano, Italy), as previously described [63,68]. Briefly, samples were incubated for 20 min at room temperature, sonicated (5 cycles/30 s), and centrifuged at 13,000× *g* for 5 min. Supernatants were collected and stored at −80 °C until use. Protein samples containing an equal amount of proteins (20 μg) were electrophoresed on 4−20% SDS-PAGE gels and transferred to nitrocellulose membranes. Membranes were incubated for 1 h at room temperature with blocking buffer (5% non-fat milk in 0.1% tween-20 in PBS) and then overnight at 4 °C with primary antibodies diluted in blocking buffer. The following primary antibodies were used for immunoblotting: Mouse anti-ChAT (Immunological Sciences, Roma, Italy; Cat.#: MAB10838; dilution: 1:400), mouse anti-GFAP (Immunological Sciences, Cat.#: MAB16117; dilution: 1:600), mouse anti-GluR1 (Santa Cruz Biotechnology Inc., Dallas, TX, USA; Cat.#: sc-12152; dilution 1:300), goat anti-GluR4 (Santa Cruz Biotechnology Inc., Cat.#: sc7614; dilution: 1:300), and mouse anti-actin (Merck-Millipore, Milano, Italy; Cat.#: MAB1501; dilution: 1:700). Then, membranes were washed 3 times in 0.1% tween-20 in PBS and then incubated for 1 h at room temperature with the appropriate secondary antibody: Goat anti-mouse (Pierce Biotechnology Inc., Thermo Fisher Scientific, Waltham, MA, USA; Cat.#: 1858413; dilution: 1:5000) or rabbit anti-goat (Merck-Millipore, Cat.#: AP106P; dilution: 1:10,000) HRP-conjugated. Proteins bands were detected with West Dura Extended Duration HRP Substrate (Thermo Fisher Scientific, Waltham, MA, USA) according to the manufacturer’s instructions and revealed with the Uvitec Cambridge Imaging System. The density of each band was quantified using ImageJ analysis software and the band density was normalized to the actin optical density measured in the same membrane.

### 4.7. Statistical Analysis

All statistical tests were performed in GraphPad Prism (version 5.00 for Mac, GraphPad Software, San Diego, CA, USA) or RStudio (version 1.0.153, RStudio Inc., Boston, MA, USA). Data were tested for normality using a D’Agostino and Pearson omnibus normality test and subsequently assessed for the homogeneity of the variance. Data that passed both tests were further analyzed by two-tailed unpaired Student’s *t*-test for comparisons of *n* = 2 groups. Comparisons of *n* > 2 groups were performed using a one-way ANOVA and Holm-Sidak’s multiple comparisons test. Principal components analysis (PCA), explained variances and the quality of representation of the variables on the factor map expressed as square cosine (Cos2) are shown, PCA are expressed as the biplot of variables and key colored arrows representing the variables; contribution to PCs. Analysis were made in RStudio using GFAP, ChAT, GluR1, GluR4, and recovery index (RI) data. For all statistical tests, *p*-values < 0.05 were considered statistically significant.

## 5. Conclusions

In conclusion, this work confirms that the use of a neurotoxic model of selective MN depletion could provide novel information about pathogenic mechanisms that are likely similar to that of MN diseases. Unlike the classical genetic models of disease, this is a valuable tool to study compensatory events in both spinal neurons and denervated muscle after a focal MN depletion, in the absence of other concurrent pathogenic factors. Interestingly, the possible use of this tool to selectively remove MNs in animals lacking or overexpressing specific genes would provide invaluable additional information about these processes. Moreover, this tool allows the modulation of the severity and sites of MN degeneration by choosing different CTB-Sap doses and injection sites, as well as the choice of an appropriate disease onset in relation to a specific experimental setting.

## Abbreviations

ALS	Amyotrophic lateral sclerosis
CLN	Centrally located nuclei
ChAT	Choline acetyltransferase
Cos2	Square cosine
CTB-Sap	Cholera toxin-B saporin
Dpl	Day post lesion
EMG	Electromyography
FUS	Fused in sarcoma
GFAP	Glial fibrillary acidic protein
GM	Gastrocnemius muscle
HC	Healthy control
MN	Motoneurons
PCA	Principal component analysis
RI	Recovery index
ROI	Region of interest
SC	Spinal cord
SMA	Spinal muscular atrophy
SOD1	Superoxide dismutase 1
TDP-43	TAR-DNA binding protein
